# Relevance of Vitamin D Receptor Target Genes for Monitoring the Vitamin D Responsiveness of Primary Human Cells

**DOI:** 10.1371/journal.pone.0124339

**Published:** 2015-04-13

**Authors:** Maja Vukić, Antonio Neme, Sabine Seuter, Noora Saksa, Vanessa D. F. de Mello, Tarja Nurmi, Matti Uusitupa, Tomi-Pekka Tuomainen, Jyrki K. Virtanen, Carsten Carlberg

**Affiliations:** 1 School of Medicine, Institute of Biomedicine, University of Eastern Finland, Kuopio, Finland; 2 Institute of Public Health and Clinical Nutrition, University of Eastern Finland, Kuopio, Finland; Roswell Park Cancer Institute, UNITED STATES

## Abstract

Vitamin D_3_ has transcriptome- and genome-wide effects and activates, via the binding of its metabolite 1α,25-dihydroxyvitamin D_3_ to the transcription factor vitamin D receptor (VDR), several hundred target genes. Using samples from a 5-month vitamin D_3_ intervention study (VitDmet), we recently reported that the expression of 12 VDR target genes in peripheral blood mononuclear cells (PBMCs) as well as 12 biochemical and clinical parameters of the study participants are significantly triggered by vitamin D_3_. In this study, we performed a more focused selection of further 12 VDR target genes and demonstrated that changes of their mRNA expression in PBMCs of VitDmet subjects significantly correlate with alterations of 25-hydroxyvitamin D3 serum levels. Network and self-organizing map analysis of these datasets together with that of the other 24 parameters was followed by relevance calculations and identified changes in parathyroid hormone serum levels and the expression of the newly selected genes *STS*, *BCL6*, *ITGAM*, *LRRC25*, *LPGAT1* and *TREM1* as well as of the previously reported genes *DUSP10* and *CD14* as the most relevant parameters for describing vitamin D responsiveness *in vivo*. Moreover, parameter relevance ranking allowed the segregation of study subjects into high and low responders. Due to the long intervention period the vitamin D response was not too prominent on the level of transcriptional activation. Therefore, we performed in the separate VitDbol trial a short-term but high dose stimulation with a vitamin D3 bolus. In PBMCs of VitDbol subjects we observed direct transcriptional effects on the selected VDR target genes, such as an up to 2.1-fold increase already one day after supplementation onset. In conclusion, both long-term and short-term vitamin D_3_ supplementation studies allow monitoring the vitamin D responsiveness of human individuals and represent new types of human *in vivo* vitamin D_3_ investigations.

## INTRODUCTION

Vitamin D_3_ is an important endocrine compound that has genome- and transcriptome-wide effects on most human tissues and cell types [1,2]. Human skin can produce vitamin D_3_ from 7-dehydrocholesterol using energy provided by the UV-B component of sun light [3]. The most abundant vitamin D metabolite, 25-hydroxyvitamin D_3_ (25(OH)D_3_), is used as a biomarker for the vitamin D status of the human body [4]. However, the biologically most active vitamin D compound is 1α,25-dihydroxyvitamin D_3_ (1,25(OH)_2_D_3_), which functions as specific high-affinity ligand of the transcription factor VDR [5].

Although vitamin D_3_ is well-known for its role in the regulation of calcium and phosphate homeostasis and the resulting impact on bone mineralization [6], it is also involved in the control of other physiological processes, such as cellular growth, intracellular metabolism as well as innate and adaptive immunity [7,8]. Humans should aim for an optimal vitamin D status, in order to take full benefit from the potential of the pleiotropic signaling molecule. However, there are high inter-individual variations in serum 25(OH)D_3_ levels due to different exposure to natural UV-B radiation or intake of vitamin D_3_ from diet or supplements. Moreover, the vitamin D status was found to be associated with age, body mass index and (epi)genetic polymorphisms [9–11]. As a general guideline the US Institute of Medicine has recommended a serum 25(OH)D_3_ concentration of 50 nM [12], which was criticized to be too low [13]. Irrespective of the exact threshold definitions, worldwide many people are vitamin D deficient. Not only the bone health of these individuals is compromised, but they have an increased risk of developing a number of diseases, such as cancer, autoimmune disorders and various features of the metabolic syndrome [14].

Measuring the biomarker serum 25(OH)D_3_ concentration is easy and straightforward, but this approach ignores the possibility that human individuals display a personalized response to vitamin D. Instead of debating about the most appropriate 25(OH)D_3_ threshold level, we suggest that human individuals should be tested for their vitamin D responsiveness. Since vitamin D_3_ is rapidly converted to 1,25(OH)_2_D_3_, which in turn directly activates the nuclear receptor VDR, expression changes of VDR target genes can serve as direct measures for the desired functional consequences of a vitamin D responsiveness. As representatives of the very mobile innate and adaptive immune system, we consider the mixture of monocytes, T and B lymphocytes, referred to as PBMCs, as the most suited tissue for our investigations. These cells can be obtained with minimal harm to the study participants and are quickly isolated.

Transcriptome-wide analysis indicated that, depending on the cell type, more than 1,000 genes change their mRNA expression after a 24 h stimulation with 1,25(OH)_2_D_3_ [15–19]. On the genome-wide level, the method chromatin immunoprecipitation coupled with massive parallel sequencing (ChIP-seq) allows the mapping of nuclear proteins, such as VDR [20]. At present, VDR ChIP-seq data are available only from cell line models, such as the B lymphocyte lines GM10855 and GM10861 [15], THP-1 monocyte-like cells [16], and lipopolysaccharide (LPS)-polarized macrophage-like THP-1 cells [21]. Although results from the FANTOM 5 project [22] question the possibility of a reliable extrapolation of genomic and transcriptomic data from cell lines to primary cells, we used our genomic insight on the high conservation of a limited number of genomic VDR binding loci [21].

In this study, we took advantage of PBMC samples and related biochemical and clinical parameters that were available from the start and the end of the vitamin D_3_ intervention trial VitDmet [23,24]. During 5 months of the Finnish winter season the VitDmet subjects had been supplemented daily with 0, 40 or 80 μg vitamin D_3_. In a previous study [25], we tested the 12 VDR target genes ArfGAP with SH3 domain, ankyrin repeat and PH domain 2 (*ASAP2*), cathelicidin anti-microbial peptide (*CAMP*), CD14 molecule (*CD14*), CD97 molecule (*CD97*), dual specificity phosphatase 10 (*DUSP10*), G0/G1switch 2 (*G0S2*), interleukin 8 (*IL8*), leucine rich repeat containing 8 family, member A (*LRRC8A*), ninjurin 1 (*NINJ1*), nuclear receptor interacting protein 1 (*NRIP1*), solute carrier family 37, member 2 (*SLC37A2*) and thrombomodulin (*THBD*) as possible markers for the vitamin D responsiveness of VitDmet study participants. Moreover, from 200 biochemical and clinical parameters that had been determined for all subjects of the VitDmet study [23], we found 12 to correlate highly significantly (r^2^ > 0.48) in their changes with the alternations of the individual's 25(OH)D_3_ serum levels. These parameters were heart rate, lymphocyte number, the serum concentration of the proteins parathyroid hormone (PTH), interleukin 6 (IL6), adiponectin (ADIPOQ), glutamic-pyruvate transaminase (GTP) and the soluble tumor necrosis factor receptor superfamily, member 1B (TNFRSF1B) and changes in the oral glucose tolerance test results at the start and end of the intervention, such as the concentrations of insulin (INS) at 0 min and free fatty acids (FFAs) at 0 min and 120 min as well as the calculated indices homeostatic modeling assessment insulin resistance (HOMA-IR) and insulin sensitivity.

Based on a more stringent evaluation, we selected for this study the 12 additional VDR target genes steroid sulfatase, isozyme S (*STS*), B-cell CLL/lymphoma 6 (*BCL6*), integrin, alpha M (*ITGAM*), leucine rich repeat containing 25 (*LRRC25*), lysophosphatidylglycerol acyltransferase 1 (*LPGAT1*), triggering receptor expressed on myeloid cells 1 (*TREM1*), CD274 molecule (*CD274*), fucosidase, alpha-L-1 (*FUCA1*), nuclear factor, erythroid 2 (*NFE2*), CD38 molecule (*CD38*), fructose-1,6-bisphosphatase 1 (*FBP1*) and transmembrane protein 37 (*TMEM37*). With all 36 vitamin D_3_-triggered parameters we conducted network and self-organizing map analysis. In addition, we used samples of a short-term but high dose (once 2,000 μg) stimulation of human individuals with vitamin D_3_ (VitDbol trial) and observed direct transcriptional effects on the selected VDR target genes already one day after supplementation onset. Both long-term and short-term vitamin D_3_ supplementation studies allow monitoring the vitamin D responsiveness of human individuals and represent a new type of human *in vivo* vitamin D_3_ investigations.

## MATERIALS AND METHODS

### VitDmet and VitDbol study samples

The participants of the VitDmet study (NCT01479933, ClinicalTrials.gov) were i) men and women ≥ 60 years of age from the region of Kuopio, Finland (63 N) [24], ii) had a starting serum 25(OH)D_3_ level of below 75 nM, iii) showed evidence of disturbed glucose homeostasis, such as impaired fasting glucose or impaired glucose tolerance, but not yet manifest type 2 diabetes, and iv) had a body mass index between 25 and 35. The study was performed during 5 months of the Finnish winter season, i.e. in a period when there is no natural production of vitamin D_3_ in the skin of the participants. In contrast, the subjects of the VitDbol study (NCT02063334) were men and women < 50 years of age, ii) showed no evidence of disturbed glucose homeostasis, and iii) had a body mass index between 20 and 25. PBMC mRNA was available for 71 VitDmet subjects at two time points (months 0 and 5) and for 10 VitDbol subjects at up to three time points (days 0, 1 and 2). The research ethics committee of the Northern Savo Hospital District had approved both study protocols (#37/2011 and #9/2014). All participants gave a written informed consent to participate in the study.

Serum concentrations for 25(OH)D_3_ were measured from venous blood samples using a high performance liquid chromatography with coulometric electrode array as described previously [26]. Moreover, for VitDmet subjects serum protein levels for the bone health marker PTH (intact protein), the cytokine receptor TNFRSF1B, the cytokine IL6, the adipocyte cytokine ADIPOQ and the liver enzyme GTP were determined by standard methods as described previously [26]. Other biochemical parameters were assayed at a local laboratory service provider (ISLAB, Kuopio, Finland). In addition, at the start and the end of the VitDmet study a 2 h oral glucose tolerance test was carried out with 75 g glucose and three time points (0, 30 and 120 min) glucose, FFAs and INS were measured. HOMA-IR indices were computed according to the nonlinear function using the approach of Wallace *et al*. [27], while the insulin sensitivity index was calculated as described by Matsuda and DeFronzo [28].

### qPCR from PBMC samples

For the VitDmet study, PBMCs were isolated at months 0 and 5, RNA was extracted and cDNA synthesized as described previously [24,29]. For the VitDbol study, PBMCs were isolated at days 0, 1 and 2, RNA was extracted with the High Pure RNA isolation kit (Roche) or the Direct-zol kit (Zymo Research) and cDNA was synthetized using the Transcriptor First Strand cDNA kit (Roche). qPCR reactions were performed using 250 nM of reverse and forward primers (S1 Table), diluted cDNA template and LightCycler 480 SYBRGreen I Master mix (Roche). In the PCR reaction the hotstart Taq polymerase was activated for 10 min at 95°C, followed by 42 amplification cycles of 20 s denaturation at 95°C, 15 s annealing at primer-specific temperatures (S1 Table) and 15 s elongation at 72°C and a final elongation for 10 min at 72°C. PCR product specificity was monitored using post-PCR melt curve analysis. Relative mRNA expression levels were determined using the formula 2^-(ΔCt)^, where ΔCt is Ct_(target gene)_—mean of Ct_(reference genes)_. The four internal reference genes beta-2-microglobulin (*B2M*), glycerinaldehyde-3-phosphate-dehydrogenase (*GAPDH*), hypoxanthine phosphoribosyltransferase 1 (*HPRT1*) and ribosomal protein, large, P0 (*RPLP0*) were used as references. The stability of the expression of the reference genes was determined using the geNorm algorithm [30].

### Correlation analysis

The correlations between the changes in mRNA expression in relation to serum 25(OH)D_3_ level alterations had previously been shown to be linear [24,29]. The extent of the correlation was measured by the r^2^ value. All possible correlations were computed by testing the line, which fits best the maximum number of participants. The largest participant subgroup that provided a correlation with an r^2^ value of 0.37 or higher was selected for further analysis. Participants were removed under the biologically plausible assumption that not all are responsive to vitamin D_3_, measured as changes in either mRNA expression or clinical parameters. The identification of a preponderant direction of changes in mRNA/parameters as a function of 25(OH)D_3_ level variations allows the elucidation of a subgroup of participants that are vitamin D responders. In a typical correlation analysis, the best-fitting line would have as its slope the correlation value, but will take into account all participants, including those that do not respond to vitamin D_3_ supplementation. The filtering of the participants that do not stick to the preponderant line is an attempt to remove outliers, so as to only responders will have an impact the subsequent network analysis. Of note, the subgroups do not contain exactly the same set of study participants.

The network analysis is based on the fit, measured by r^2^, of the changes in mRNA expression/clinical parameter in relation to 25(OH)D_3_ serum level variations, for the participants not removed. In the network, the correlation between two genes/parameters is a measure of that fitness. This was computed by testing the participants in the filtered group for the first gene/parameter on their correlation between the changes in mRNA for a second gene or parameter and serum level variations. The higher the correlation, the thicker the line is.

### ChIP-seq data visualization

The Integrative Genomics Viewer (IGV) [31] was used to visualize the VDR ChIP-seq data, which had been summarized by Tuoresmäki *et al*. [21] at Gene Expression Omnibus (GEO, www.ncbi.nlm.nih.gov/geo) under the accession number GSE53041.

### Network construction

The Python-based software package NetworkX (https://networkx.github.io) was applied to represent the information obtained from the correlation analysis by a network graph. The latter was further processed with the open software Gephi (https://gephi.github.io), which is an interactive visualization and exploration platform for a variety of networks and complex systems, dynamic and hierarchical graphs. For self-organizing map (SOM) construction, each of the 36 investigated parameters was represented by a correlation vector to the remaining 35 parameters, i.e. each vector had 35 attributes. In order to obtain a visual impression on the similarity of the investigated parameters, their correlation data were mapped on a lattice of 25x25 units.

### Parameter relevance calculations

The relevance of each parameter was obtained from the 36x36 correlation matrix (S2 Table). For each parameter i the average correlation to all remaining 35 parameters and the proportion of pairwise correlations (r > 0.3) were calculated. The average of these two quantities defines a relevance index for parameter i based on the correlation from this to all remaining parameters. In parallel, the average correlation for each of the remaining 35 parameters to parameter i and the proportion of the pairwise correlations (r > 0.3) were calculated defining a relevance index for parameter i based on the correlation from another parameter to it. The relevance of parameter i is the average of both indices.

## RESULTS

### VDR target gene expression changes in PBMCs

Based on information provided by i) a harmonized re-analysis of VDR ChIP-seq data from the human B cell lines GM10855 and GM10861, human monocytic THP-1 cells and human macrophage-like LPS-differentiated THP-1 cells [21] and ii) a microarray of undifferentiated THP-1 cells that were treated for 24 h with 1,25(OH)_2_D_3_ [19], we selected the 12 VDR target genes *STS*, *BCL6*, *ITGAM*, *LRRC25*, *LPGAT1*, *TREM1*, *CD274*, *FUCA1*, *NFE2*, *CD38*, *FBP1* and *TMEM37* (S1 Fig) for expression studies in human PBMCs. The selection criteria for the genes were that i) they carry in distance of less than 250 kb from their transcription start site (TSS) a genomic VDR binding site that is in at least two hematopoietic cell models occupied by the receptor (summarized in S3 Table), ii) their expression in THP-1 cells was at least 2-fold induced after 24 h treatment with VDR ligand [19] and iii) they show reasonable basal expression in human PBMCs.

**Fig 1:**
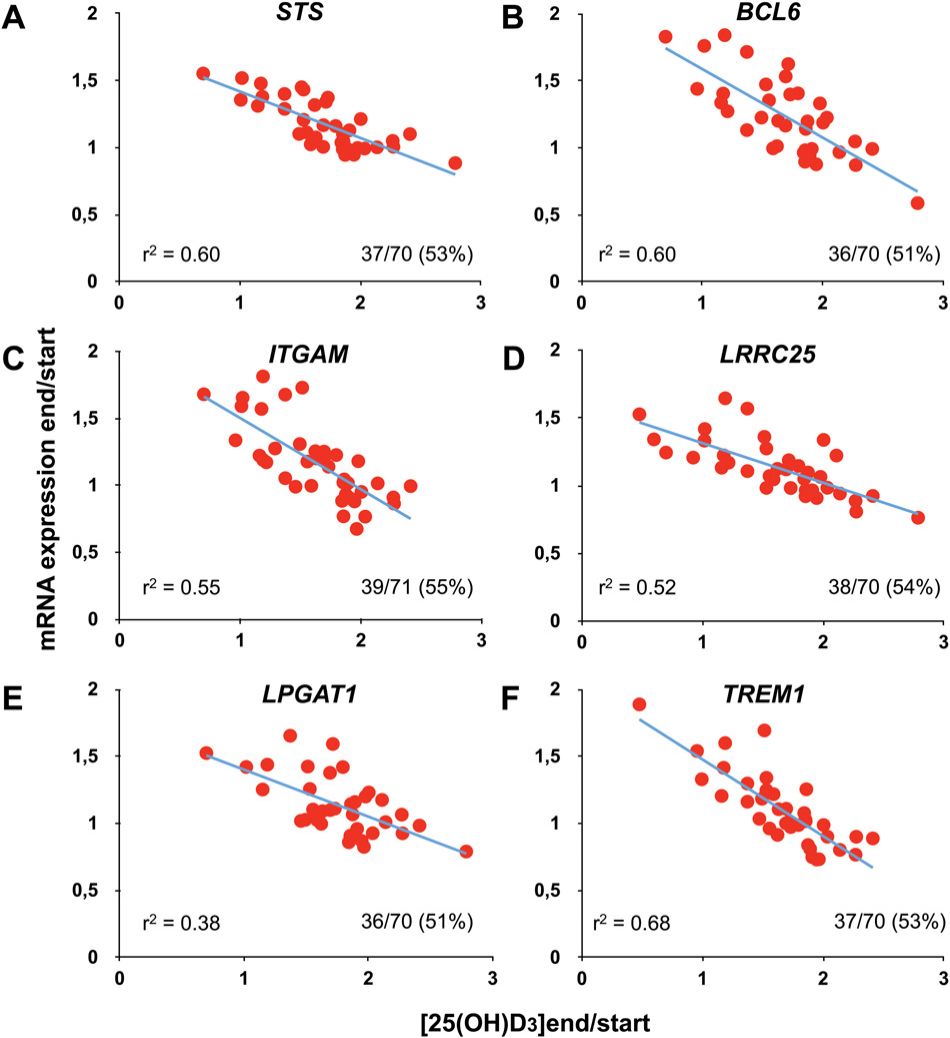
VDR target gene expression changes in PBMCs of VitDmet study participants. RNA was isolated from PBMCs obtained from 70 or 71 participants of the VitDmet study before and after the 5-month vitamin D_3_ intervention. qPCR was performed to determine the relative changes of the expression of the VDR target genes *STS* (**A**), *BCL6* (**B**), *ITGAM* (**C**), *LRRC25* (**D**), *LPGAT1* (**E**) and *TREM1* (**F**) normalized by the reference genes *B2M*, *GAPDH*, *HPRT1* and *RPLP0*. Linear regression analysis demonstrated the correlation between mRNA expression changes and alterations in the serum 25(OH)D_3_ levels of the participants. The number of selected study participants and the r^2^ correlation value are indicated.

A supplementation of VitDmet study participants with daily either 0, 40 or 80 μg vitamin D_3_, resulted in serum 25(OH)D_3_ levels between 27.5 and 155.7 nM at the end of the study [24]. This represents vitamin D status changes of the study subjects ranging from a 2.1-fold reduction to a 2.8-fold increase (Fig 1 and S2 Fig). Importantly, during the 5 months of the intervention none of the participants displayed a significant change in body mass index or in serum calcium concentration (for further details see [23,24]). RNA was isolated from PBMCs of 71 VitDmet participants at the start and the end of the trial and qPCR was used to determine the relative changes in mRNA expression (normalized with four reference genes) of the 12 selected VDR target genes during the 5 months of the intervention (Fig 1 and S2 Fig). When using data from all study participants, we could not observe any significant correlation between VDR target gene expression changes and respective 25(OH)D_3_ level alterations. Therefore, we applied a systematic correlation analysis and, assuming a linear correlation, explored all possible lines through the graphs. In order to obtain the best possible correlation, we allowed the step-wise elimination of up to 35 subjects, i.e. up to 50% of all study participants. This approach resulted in highly significant correlations (r^2^ > 0.37), when the data of 37 subjects for *STS* (Fig 1A), 36 for *BCL6* (Fig 1B), 39 for *ITGAM* (Fig 1C), 38 for *LRRC25* (Fig 1D), 36 for *LPGAT1* (Fig 1E), 37 for *TREM1* (Fig 1F), 36 for *CD274* (S2 Fig), 40 for *FUCA1* (S2 Fig), 39 for *NFE2* (S2 Fig), 40 for *CD38* (S2 Fig), 40 for *FBP1* (S2 Fig) and 37 for *TMEM37* (S2 Fig) were used. The expression of all 12 genes was shown to be up-regulated after 24 h stimulation of the cell line THP-1 with 1,25(OH)_2_D_3_ [19]. In contrast, our systematic correlation approach suggested that in PBMCs only the expression of the genes *CD38* and *TMEM37* kept a positive correlation with changes in 25(OH)D_3_ levels. This means that during the long period of 5 months *in vivo* the expression of the remaining 10 VDR target genes decreased with increasing vitamin D status of the study subjects.

In summary, the VDR target genes *STS*, *BCL6*, *ITGAM*, *LRRC25*, *LPGAT1*, *TREM1*, *CD274*, *FUCA1*, *NFE2*, *CD38*, *FBP1* and *TMEM37* i) carry a conserved VDR binding site in relative vicinity to their TSS, ii) are 1,25(OH)_2_D_3_ targets in a human monocytic cell line and iii) show well detectable expression in human PBMCs. The mRNA expression changes of the 12 genes in PBMCs significantly correlate with alterations of 25(OH)D_3_ serum levels when restricting to data from 36 to 40 (51–57%) of the VitDmet study participants.

### Network analysis of vitamin D_3_-triggered parameters

**Fig 2:**
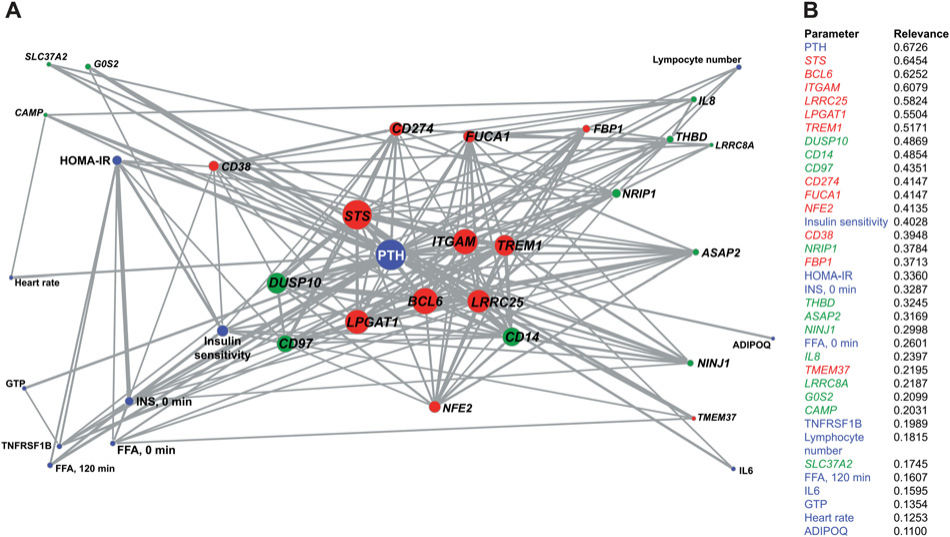
Network analysis and parameter relevance. Correlation analysis (S2 Table) of the vitamin D_3_-dependent changes in the expression of 12 newly introduced VDR target genes (red nodes), 12 previously analyzed [25] VDR target genes (green nodes) and physiological parameters (blue nodes) suggested a network (**A**). Correlations with an r value higher than 0.3 between the parameters are indicated by connection lines. The size of the nodes is proportional to the number of connections of the respective parameter. Thicker connection lines represent correlations of higher significance. The relevance ranking of the 36 evaluated parameters is represented by the font size of their labels. The 36 parameters were ranked by their relevance in describing the vitamin D_3_-triggered observed effects in samples of the VitDmet study (**B**). Clinical and biochemical parameters are shown in blue, newly reported VDR target genes in red and previously reported target genes in green.

In a previous study [25], we had already identified the 12 VDR target genes as well as 12 biochemical and clinical parameters to correlate highly significantly (r^2^ > 0.48) in their changes with the alternations of the VitDmet study subject's 25(OH)D_3_ serum levels (gray 24x24 matrix in S2 Table). In order to investigate, whether these 24 vitamin D_3_-triggered parameters and the 12 new VDR target genes described above, influence each other and form a correlation network, we combined them in a 36x36 correlation matrix (represented by the square root of r^2^, S2 Table). Connection lines in the correlation network (Fig 2A) indicate that responders concerning one parameter can be fitted significantly (r > 0.3) to other parameter, to which the line is directing. The number of connections and their significance allows sorting of the 36 parameters by their relevance (Fig 2B). Serum PTH concentration was found to be the most relevant parameter, which fits well with its role of an established biomarker for bone health [32]. Importantly, the mRNA expression changes of the newly reported VDR genes *STS*, *BCL6*, *ITGAM*, *LRRC25*, *LPGAT1* and *TREM1* (Fig 1) were found next in line as most relevant parameters for describing the vitamin D_3_-triggered response of VitDmet study participants (Fig 2B). Then the VDR target genes *DUSP10* and *CD14* are listed, which had been reported already earlier as biomarkers for the vitamin D response of VitDmet study subjects [24,25]. Together with PTH these eight genes form the center of the network (Fig 2A).

In a similar, but independent approach the relation of 36 vitamin D_3_-triggered parameters was displayed in a SOM (S3 Fig). A SOM represents a non-linear projection of high-dimensional data, such as the possible correlation of one parameter with 35 other parameters, into a low-dimensional space, such as 2-dimensional map. Closely related parameters, such as PTH and the eight above-listed genes are found in the same lower left corner of the SOM and were separated from the other parameters by areas of dissimilarities (red units in S3 Fig).

Taken together, at present we tested in total 36 parameters, such as gene expression changes of 24 VDR target genes and the alterations of 12 clinical and biochemical values, which were significantly triggered by vitamin D_3_. Network and SOM analysis as well as relevance calculations allowed the identification of PTH serum levels and the expression of the genes *STS*, *BCL6*, *ITGAM*, *LRRC25*, *LPGAT1*, *TREM1*, *DUSP10* and *CD14* as the most relevant parameters for describing the vitamin D response *in vivo*.

### Segregating high from low vitamin D responders

**Fig 3:**
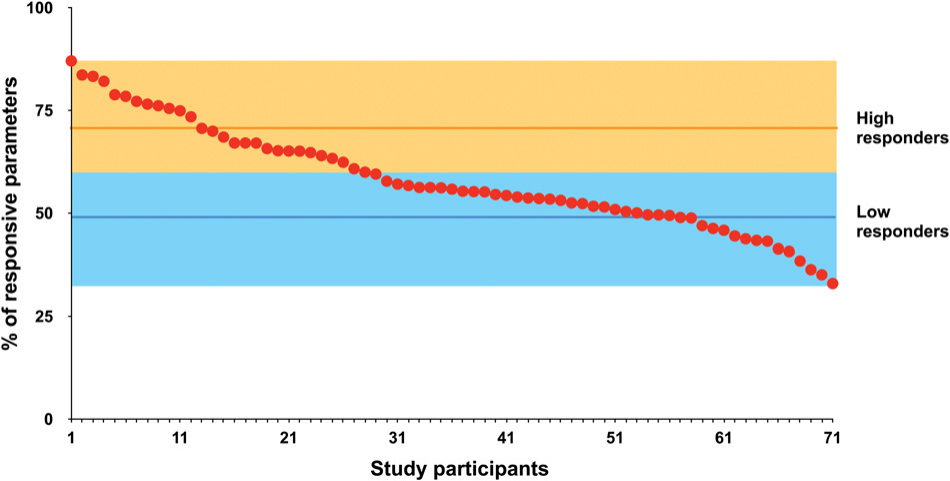
High and low responders of the VitDmet study. The vitamin D_3_ response of the 71 VitDmet study participants is expressed by the percentage (red data points) of the 36 vitamin D_3_-triggered parameters, to which they show response. Each parameter was weighted by its individual relevance coefficient (Fig 2B). A k-mean approach was used to distinguish 26 high responders (orange) from 45 low responders (blue). The horizontal lines indicate the mean values of the two groups.

For each of the 71 VitDmet study participants we determined the number of the parameters, ranging from 10 to 30 out of 36, to which the individual showed responsiveness. The parameters were weighted by their relevance coefficient (Fig 2B) and their sum is expressed as percentage of the maximal sum (Fig 3). We applied the k-means algorithm, in order to segregate high from low responders. This identified 26 persons in the group of high responders and 45 in the low responder group.

We next evaluated how many parameters needed to be measured, in order to accurately segregate high from low responders. In the order of parameter relevance ranking (Fig 2B), i.e. starting with PTH serum levels, we added one by one further parameters and determined the number of mismatches to the VitDmet participant classification based on all 36 parameters (S4 Fig). This suggested that the first 17 parameters should be used, in order to obtain a reasonably accurate (less than 5% mismatches) classification. For the determination of the 14th parameter in the ranking, insulin sensitivity, an oral glucose tolerance test has to be performed at the start and end of the study. Since this involves more effort than measuring the expression of a few additional VDR target genes by qPCR, we did the accuracy evaluation by excluding insulin sensitivity and obtained a very comparable result (S4 Fig). This means that an accurate classification is obtained by determining the PTH serum level and the expression of the 16 top-ranking VDR target genes in PBMCs.

In summary, the use of parameter relevance ranking appears to be an accurate tool for the segregation of VitDmet study participants into high (26) and low (45) responders. In order to obtain sufficient accuracy of the classification, the 17 top-ranking parameters should be determined.

### Long-term versus short-term vitamin D_3_ response of PBMCs

The 5-month intervention period with vitamin D_3_ of the VitDmet study is far longer than the time needed for the enzymatic conversion of vitamin D_3_ to its biologically active form, 1,25(OH)_2_D_3_, and the signal transduction of the latter nuclear hormone. Both are in the order of 24 h or less [33,34]. In order to distinguish possible long-term effects of vitamin D_3_ from short-term activities, we started a new type of vitamin D_3_ intervention, VitDbol, where healthy human adults were supplemented once with a high dose of vitamin D_3_ (2,000 μg) and PBMCs were isolated at days 0, 1 and 2. We selected 10 subjects of the VitDbol study, for which PBMC RNA was available, for further analysis. The individual's starting 25(OH)D_3_ levels ranged from 37.3 to 101.7 nM and increased at day 1 already up to 1.43-fold and at day 2 up to 1.53-fold.

We compared PBMC samples obtained from the long-term VitDmet study with those of the short-term VitDbol study by monitoring the basal mRNA expression of the 12 most relevant VDR target genes (Fig 2B) together with that of the genes *CD38*, *FBP1*, *LRRC8A* and *CAMP* (S5 Fig). The levels of the highly expressed genes *CD14*, *LRRC25*, *CD97*, *NFE2* and *FUCA1* as well as the lower expressed genes *FBP1* and *LRRC8A* were found to be nearly identical in both types of samples. In contrast, the genes *TREM1*, *BCL6*, *LPGAT1*, *ITGAM*, *DUSP10*, *CD38*, *STS* and *CD274* are 1.6- to 4.7-fold higher expressed in PBMCs of elderly, pre-diabetic individuals than in younger healthy persons. Interestingly, the expression of the *CAMP* gene, which encodes for an anti-bacterial peptide, varied a lot in elderly VitDmet participants and showed in average 6.3-fold lower levels than in the younger VitDbol subjects.

**Fig 4:**
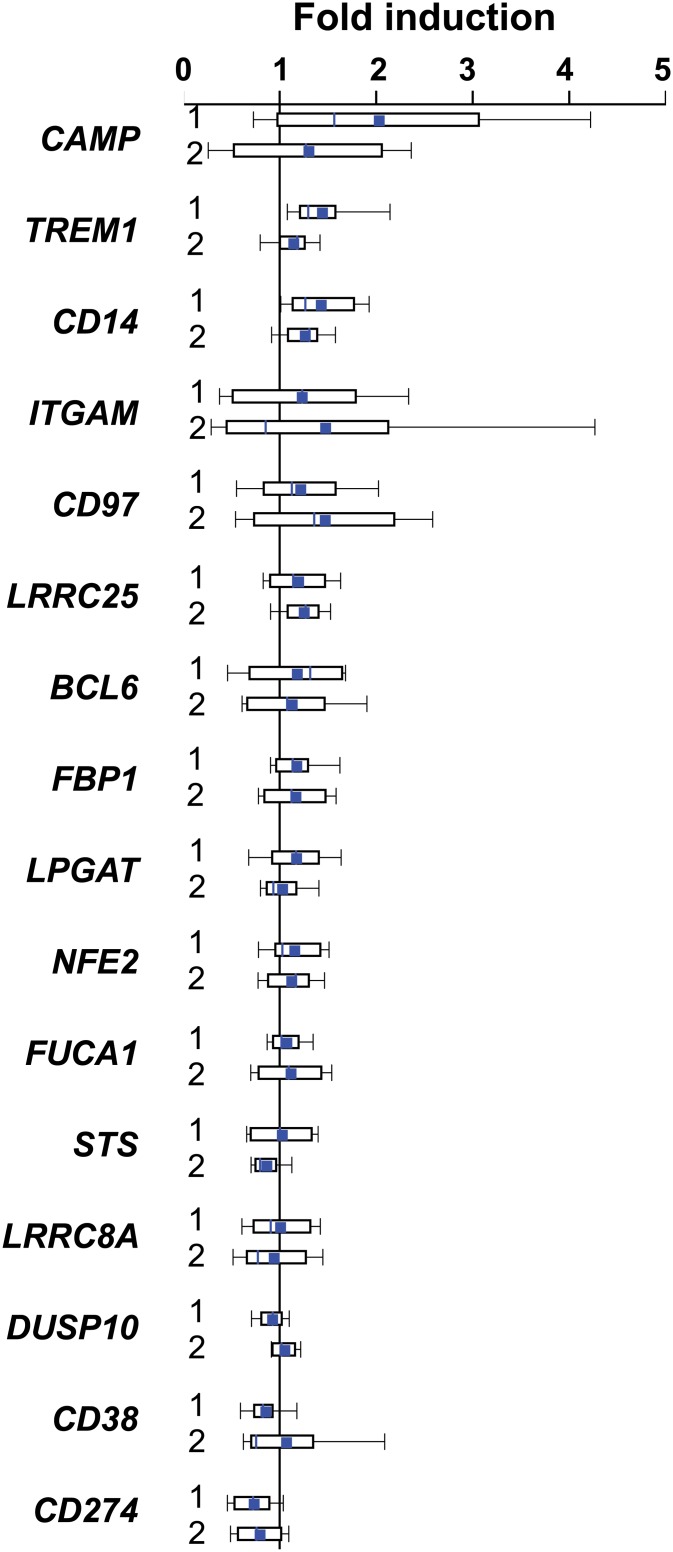
Range of mRNA expression changes in PBMCs of VitDbol study participants. qPCR was used to determine the change in mRNA expression of 16 most relevant VDR target genes in PBMCs isolated from 4–5 samples of the VitDbol study, where healthy adults were supplemented once with a high vitamin D_3_ dose (2,000 μg). Gene expression was measured at days 0, 1 and 2 and fold changes were calculated between days 1 and 0 (1) and days 2 and 0 (2). The top, fine central and bottom lines of the box plots represent the 75th, 50th and 25th percentiles, respectively, of the expression changes and the bars indicate standard deviations. The prominent blue central line within the box plots marks the means. The genes were sorted from highest to lowest induction at day 1.

Next we assessed the change of the expression of the same 16 genes 1 and 2 days after the supplementation with a high vitamin D_3_ dose (Fig 4). Interestingly, in average most tested genes were up-regulated ranging from a 2.1-fold induction of *CAMP* mRNA expression at day 1 to a 1.3-fold suppression of *CD274*. In contrast, the 5-month daily intervention with vitamin D_3_ resulted in average of all VitDmet samples only in a range of no regulation to a 1.2-fold up-regulation (e.g., for *CAMP*, S6 Fig). Moreover, in contrast to the negative correlation that most tested genes displayed in comparison of their change in mRNA expression during 5 months over the alteration of their 25(OH)D_3_ serum levels (Fig 1 and S2 Fig), only the genes *CD38* and *CD274* were slightly down-regulated in the short-term stimulated samples.

Taken together, short-term stimulations of human individuals with a high dose of vitamin D_3_, as performed in the VitDbol trial, can cause an up to 43% increase of serum 25(OH)D_3_ levels after one day and even an up to 53% increase after two days. Such *in vivo* vitamin D_3_ stimulation experiments result in more prominent effect on the mRNA expression of VDR target genes than a long-term daily supplementation with moderate amounts of vitamin D_3_, as assessed in the VitDmet study.

## DISCUSSION

In this study, we used genome- and transcriptome-wide insight, such as VDR ChIP-seq and microarray data, from human hematopoietic cell lines, to select the 12 VDR target genes for assessing of their possible vitamin D_3_-triggered regulation in human PBMCs. The primary cells were obtained from participants of two different types of vitamin D_3_ intervention studies: i) from up to 71 elderly, pre-diabetic subjects of the VitDmet study at months 0 and 5 and ii) from up to 10 younger healthy individuals of the VitDbol trial at days 0, 1 and 2. In both studies, the 25(OH)D_3_ serum levels at the start and the end showed a wide range, i.e. they well represent the naturally occurring variety in the vitamin D status of human individuals.

Network and SOM analysis, which was based on the correlation matrix of all measured 36 parameters, identified PTH serum levels as well as the expression changes of the eight genes *STS*, *BCL6*, *ITGAM*, *LRRC25*, *LPGAT1*, *TREM1*, *DUSP10* and *CD14* as the most relevant parameters for monitoring the vitamin D_3_-induced effects within VitDmet study subjects. The genes *DUSP10* and *CD14* had already been highlighted in previous studies [24,25], but the remaining, newly reported VDR target genes appear to be even more relevant than the latter.

The relevance ranking of the vitamin D_3_-triggered parameters measured from samples of VitDmet study participants allows an accurate segregation into high and low responders to vitamin D_3_. On the present stage, the top 17 parameters should be determined, in order to obtain a correct distinction between the two groups. While the top-ranking parameter, the well-established biomarker PTH serum level [32], will be routinely measured in any case, the other parameters represent the expression changes of VDR target genes in PBMCs, i.e. they can be determined rather easily in the same series of PCR experiments. Interestingly, the 26 high responders to vitamin D_3_ entered the VitDmet study with rather variant 25(OH)D_3_ serum levels ranging from 37.8 to 73.2 nM but all showed a clear functional benefit from raising their vitamin D status. This supports our thinking that rather than aiming for a general population-based optimal 25(OH)D_3_ serum level for everyone, every individual should be supplemented to meet his/her personalized needs to achieve these optimal levels.

It is remarkable that expression changes of VDR target genes in PBMCs can serve as biomarkers of the functional consequences of a vitamin D supplementation over 5 months. The latter period is far longer than the time needed for gene regulation by vitamin D as well as the life span of PBMCs. Thus, it is no surprise that in average of all VitDmet participants the mRNA expression of the tested VDR target genes increases not more than 1.2-fold. Moreover, the analysis of the mRNA expression changes in relation to alterations of 25(OH)D_3_ serum levels suggested for most VDR target genes a negative correlation. This would imply that the expression of these genes should decrease with an increasing vitamin D status, although all genes have been selected as being up-regulated by vitamin D. In the THP-1 human monocytic cell line model, we recently observed that 1,25(OH)_2_D_3_ modulates, via activities of its nuclear receptor VDR, the accessibility of genomic DNA by opening and closing chromatin [35,36]. This applies also to primary cells, such as PBMCs (S.S., A.N., C.C., unpublished results). Therefore, we assume that the observed functional consequences of vitamin D_3_ supplementation on VDR target gene expression in PBMCs of participants of the VitDmet study are rather on the level of epigenetics rather than direct effects on transcription.

In contrast, with samples from the short-term, high dose vitamin D_3_ supplementation study VitDbol we could demonstrate a direct effect of vitamin D on the transcriptional activation of VDR target genes. The single 2,000 μg vitamin D_3_ dose raised the 25(OH)D_3_ serum level within one day by up to 43% and resulted in an up to 2.1-fold induction of VDR target gene expression. Importantly, in VitDbol PBMC samples with the exception of *CD38* and *CD274* all tested VDR target genes are up-regulated. This may due to a biphasic response, where most of the genes get up-regulated in short-term but weeks or months later they are down-regulated. Measurements at additional time points will be necessary, in order test this hypothesis. Concerning the main question of this study, the monitoring of functional consequences of vitamin D_3_ supplementations, the approach of VitDbol seems to be far more straightforward. First results can be obtained one or two days after onset of supplementation and allow the individualized adaption of the vitamin D_3_ supplementation protocol. Thus the daily vitamin D_3_ doses will be chosen to keep the individual on his/her optimal vitamin D status.

In conclusion, in this study we demonstrated that the vitamin D responsiveness of primary human cells, such as PBMCs, can be monitored via carefully selected VDR target genes. This approach is possible with samples from both long-term and short-term vitamin D_3_ intervention studies. However, the results obtained from long-term studies are probably rather on the epigenetic than on the transcriptional level. Nevertheless, a stimulation of human cohorts with a safe gene regulatory compound, such as vitamin D_3_, and the analysis of primary cells directly after their isolation, i.e. avoiding *ex vivo* culture, represents a new type of human *in vivo* experiments.

## Supporting Information

S1 Table Reverse transcription qPCR primers. (PDF)

S2 Table Correlation analysis matrix. For the 71 participants of the VitDmet study correlation analysis was performed between mRNA expression of the VDR genes introduced in this study as well as that of previously reported VDR target genes and of vitamin D_3_-triggered physiological parameters, i.e. the sub-matrix underlayed in gray represents already published data [25]. The results are indicated as square root of r^2^ values. The data shown in Fig 1 and S2 Fig are shaded in red. The network (Fig 2) displays correlations with r > 0.3 (shaded in green).
(XLSX)

S3 Table VDR target gene summary. For the 12 selected VDR target genes (see S1 Fig) the TSS position, the VDR peak position and their distance (negative numbers indicate upstream location) are indicated. Moreover, information about the conservation of the VDR peak in the four cellular models and the presence of a DR3-type VDR binding site below the peak summit (+/- 100 bp) is provided based on our recent harmonized analysis of VDR ChIP-seq datasets [21].
(PDF)

S1 Fig Genomic view on VDR target genes. The IGV browser was used to display normalized VDR ChIP-seq signals from unstimulated (-) and ligand-stimulated (+) lymphoblastoid cell lines GM10855 ([15], dark blue) and GM10861 ([15], light blue), undifferentiated THP-1 cells ([16], red) and LPS-differentiated THP-1 cells ([21], orange) for the loci of the genes *STS* (**A**), *BCL6* (**B**), *ITGAM* (**C**), *LRRC25* (**D**), *LPGAT1* (**E**), *TREM1* (**F**), *CD274* (**G**), *FUCA1* (**H**), *NFE2* (**I**), *CD38* (**J**), *FBP1* (**K**) and *TMEM37* (**L**). Gene structures are indicated in blue. The orientation of the VDR target gene and the position of its TSS are indicated by an arrow.
(PDF)

S2 Fig VDR target gene expression changes in PBMCs of VitDmet study participants. RNA was isolated from PBMCs obtained from 70 or 71 participants of the VitDmet study before and after the 5-month vitamin D_3_ intervention. qPCR was performed to determine the relative changes of the expression of the VDR target genes *CD274* (**A**), *FUCA1* (**B**), *NFE2* (**C**), *CD38* (**D**), *FBP1* (**E**) and *TMEM37* (**F**) normalized by the reference genes *B2M*, *GAPDH*, *HPRT1* and *RPLP0*. Linear regression analysis demonstrated the correlation between mRNA expression changes and alterations in the serum 25(OH)D_3_ levels of the participants. The number of selected study participants and the r^2^ correlation value are indicated.
(PDF)

S3 Fig SOM analysis. The correlation of the vitamin D_3_-dependent changes in the expression of 12 newly introduced VDR target genes, 12 previously analyzed [25] VDR target genes and physiological parameters is represented by a SOM of 25x25 units. The most relevant parameters cluster in the lower left corner of the map. The color of each unit is associated to the similitude of the surrounding units ranging from red (dissimilar) to blue (similar).
(PDF)

S4 Fig Parameter number evaluation. Starting in the order of the parameter relevance ranking (Fig 2B) the number of mismatches of segregating VitDmet participants into high and low responders was determined when increasing the number of used parameters (in reference to the classification shown in Fig 3). The first 17 parameters have to be included for a sufficiently accurate classification of the subjects. The analysis was done by including insulin sensitivity as 14th parameter (red) or by excluding it (blue).
(PDF)

S5 Fig Basal mRNA expression in PBMCs. qPCR was used to determine relative mRNA expression of 16 most relevant VDR target genes in PBMCs isolated from 70 or 71 elderly pre-diabetic subjects at the begin of VitDmet study (red) or from 10 healthy adult subjects at the start of the of the VitDbol study (blue). Data points represent the means of gene expression and the bars indicate standard deviations. The genes were sorted by decreasing basal mRNA expression in VitDbol participants.
(PDF)

S6 Fig Range of mRNA expression changes in PBMCs of VitDmet study participants. qPCR was used to determine the change in mRNA expression of 16 most relevant VDR target genes in PBMCs isolated from 70 or 71 elderly pre-diabetic subjects at the begin and the end of the 5-month vitamin D_3_ intervention trial (VitDmet). The top, fine central and bottom lines of the box plots represent the 75th, 50th and 25th percentiles, respectively, of the expression changes and the bars indicate standard deviations. The prominent blue central line within the box plots marks the means. The genes were presented in the same order as in Fig 4.
(PDF)
