# Supplementary material for: Relevance of Vitamin D Receptor Target Genes for Monitoring the Vitamin D Responsiveness of Primary Human Cells
Source: PLoS One. 2015 Apr 13;10(4):e0124339. doi: 10.1371/journal.pone.0124339 (PMC4395145; doi:10.1371/journal.pone.0124339)
Supplement: S1 Table — (PDF) [file pone.0124339.s001.pdf]

Table S1: Reverse transcription qPCR primers.

Gene	Fragment size (bp)	Annealing temperature (°C)	Primer sequences (5’-3’)
BCL6	231	62	AGAGCCCATAAAACGGTCCT<br>AGTGTCCACAACATGCTCCA
B2M[1]	246	60	GGCTATCCAGCGTACTCCAAA<br>CGGCAGGCATACTCATCTTTTT
CAMP	135	60	CAGCAGTCACCAGAGGATTGT<br>CAGCAGGGCAAATCTCTTGTTA
CD14	142	60	ACGCCAGAACCTTGTGAGC<br>GCATGGATCTCCACCTCTACTG
CD274	150	60	TGCCGACTACAAGCGAATTACTG<br>CTGCTTGTCCAGATGACTTCGG
CD38	193	60	CAACTCTGTCTTGGCGTCAGT<br>CCCATACACTTTGGCAGTCTACA
CD97	133	60	GGGACAAGAACGTCACTATGG<br>GCCAGCAATGTCGTCATGT
DUSP10	183	60	GCGGCAGTACTTTGAAGAGG<br>ATTGGTCGTTTGCCTTTGAC
FBP1	102	60	AAACACGCCATCATAGTGGAAC<br>TCCAACGGACACAAGGCAATC
FUCA1	195	60	TCCTGTCACCATGGAGGATAC<br>GTTGCCTCCCAAACTTACTGTC
GAPDH[1]	133	60	CATGAGAAGTATGACAACAGCCT<br>AGTCCTTCCACGATACCAAAGT
HPRT1[1]	94	60	TGACACTGGCAAAACAATGCA<br>GGTCCTTTTCACCAGCAAGCT
ITGAM	128	60	CTTAATACCATCGCATCCAAG<br>CTTCCTGTCTGAGTACCCTC
LPGAT1	281	62	TGTTACTCTGCCAAGGTCTGG<br>GCCAAGTGGTAAGGTCATCAG
LRRC8A	205	60	CCACCCAGCTCTTCTACTGC<br>AGTGACTGCAGCACGTTGTT
LRRC25	148	60	CTCCACTCCCGACTATGAGAAC<br>GTTACAGTAGACAGGCTGGGAAG
NFE2	158	64	GGAGAGATGGAACTGACTTGGC<br>GAATCTGGGTGGATTGAGCAGG
RPLP0[1]	318	60	AGATGCAGCAGATCCGCAT<br>GTGGTGATACCTAAAGCCTG
STS	176	60	CCGCACTGGAGTTTTCCTCT<br>AGCCGTGATGTAAAGGGTGG
TMEM37	147	64	TGGAGTTCCTCATGGTGTCCCA<br>GTGTGACTTGGTTCCTGAGGAG
TREM1	133	60	TGCTGTGGATGCTCTTTGTCT<br>TTCTGGCTGCTGGCAAACTT

[1] reference gene
