# Supplementary material for: Relevance of Vitamin D Receptor Target Genes for Monitoring the Vitamin D Responsiveness of Primary Human Cells
Source: PLoS One. 2015 Apr 13;10(4):e0124339. doi: 10.1371/journal.pone.0124339 (PMC4395145; doi:10.1371/journal.pone.0124339)
Supplement: S3 Table — For the 12 selected VDR target genes (see S1 Fig) the TSS position, the VDR peak position and their distance (negative numbers indicate upstream location) are indicated. Moreover, information about the conservation of the VDR peak in the four cellular models and the presence of a DR3-type VDR binding site below the peak summit (+/- 100 bp) is provided based on our recent harmonized analysis of VDR ChIP-seq datasets [21]. (PDF) [file pone.0124339.s003.pdf]

**Table S3: VDR target gene summary.**

| Gene | Chr | TSS position | VDR peak summit position | Distance peak-TSS | Peak GM10855? | Peak GM10861? | Peak THP-1? | Peak THP-1, LPS? | DR3? |
|---|---|---|---|---|---|---|---|---|---|
| ***STS*** | X | 7,137,472 | 7,002,259 | -135,213 | yes | yes | yes | yes | no |
| ***BCL6*** | 3 | 187,454,285 | 187,529,554 | -75,269 | yes | yes | yes | yes | yes |
| ***ITGAM*** | 16 | 31,271,288 | 31,265,138 | -6,150 | no | no | yes | yes | no |
| ***LRRC25*** | 19 | 18,508,415 | 18,613,576 | -105,161 | yes | yes | yes | yes | yes |
| ***LPGAT1*** | 1 | 212,004,114 | 212,106,338 | -102,224 | yes | yes | yes | yes | yes |
| ***TREM1*** | 6 | 41,254,457 | 41,040,849 | 213,608 | yes | yes | yes | no | no |
| ***CD274*** | 9 | 5,450,503 | 5,558,493 | 107,990 | yes | yes | yes | yes | yes |
| ***FUCA1*** | 1 | 24,194,859 | 24,269,666 | -74,807 | yes | yes | no | no | yes |
| ***NFE2*** | 12 | 54,694,821 | 54,698,280 | -3,459 | no | no | yes | yes | no |
| ***CD38*** | 4 | 15,779,931 | 15,756,115 | -23,816 | yes | yes | yes | yes | yes |
| ***FBP1*** | 9 | 97,401,923 | 97,411,633 | -9,710 | no | no | yes | yes | yes |
| ***TMEM37*** | 2 | 120,189,446 | 120,184,525 | -4,921 | no | no | yes | yes | yes |
