# Supplementary figures and images for: Relevance of Vitamin D Receptor Target Genes for Monitoring the Vitamin D Responsiveness of Primary Human Cells
Source: PLoS One. 2015 Apr 13;10(4):e0124339. doi: 10.1371/journal.pone.0124339 (PMC4395145; doi:10.1371/journal.pone.0124339)

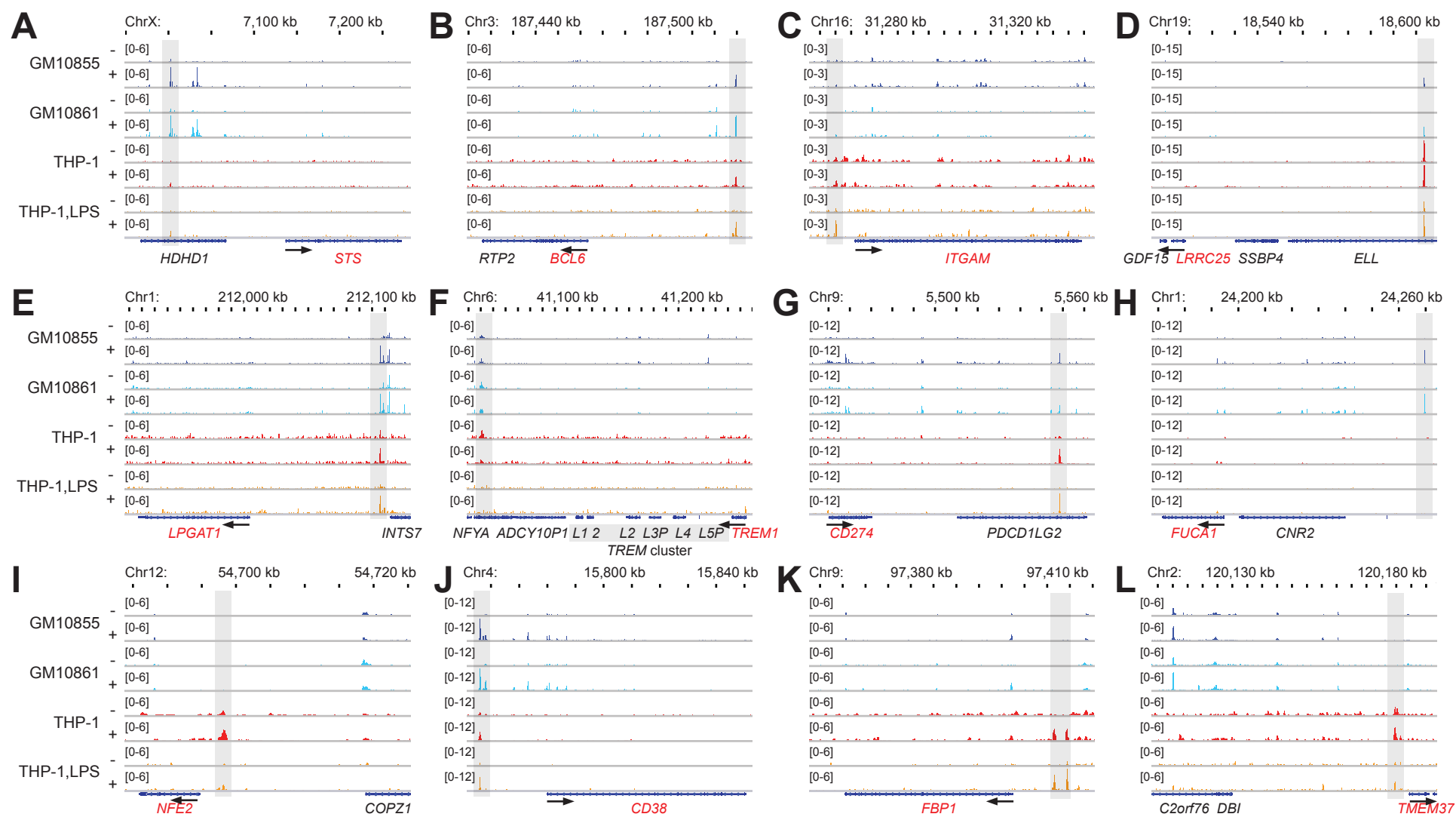

Supplement: S1 Fig — The IGV browser was used to display normalized VDR ChIP-seq signals from unstimulated (-) and ligand-stimulated (+) lymphoblastoid cell lines GM10855 ([15], dark blue) and GM10861 ([15], light blue), undifferentiated THP-1 cells ([16], red) and LPS-differentiated THP-1 cells ([21], orange) for the loci of the genes STS (A), BCL6 (B), ITGAM (C), LRRC25 (D), LPGAT1 (E), TREM1 (F), CD274 (G), FUCA1 (H), NFE2 (I), CD38 (J), FBP1 (K) and TMEM37 (L). Gene structures are indicated in blue. The orientation of the VDR target gene and the position of its TSS are indicated by an arrow. (PDF) [file pone.0124339.s004.pdf]

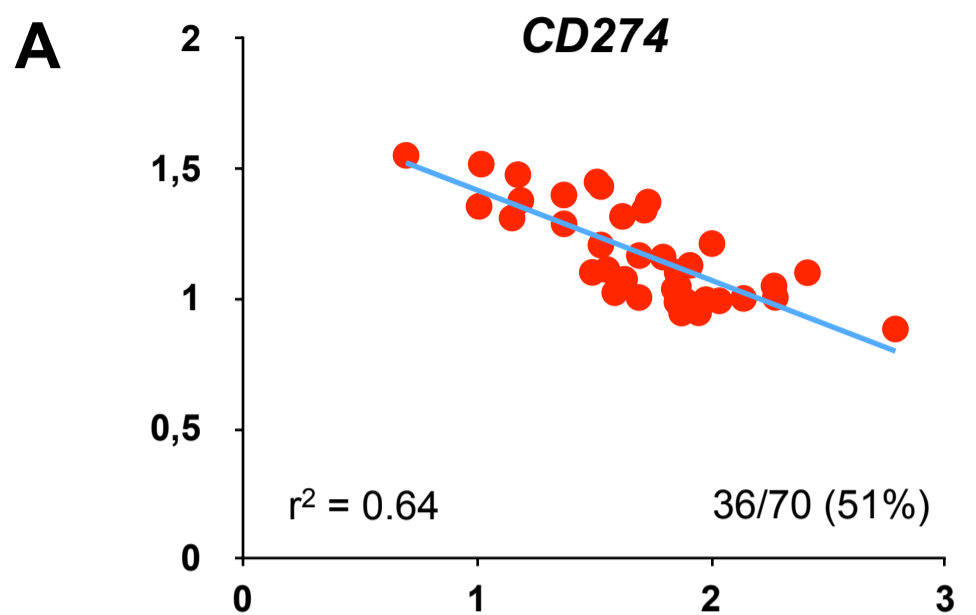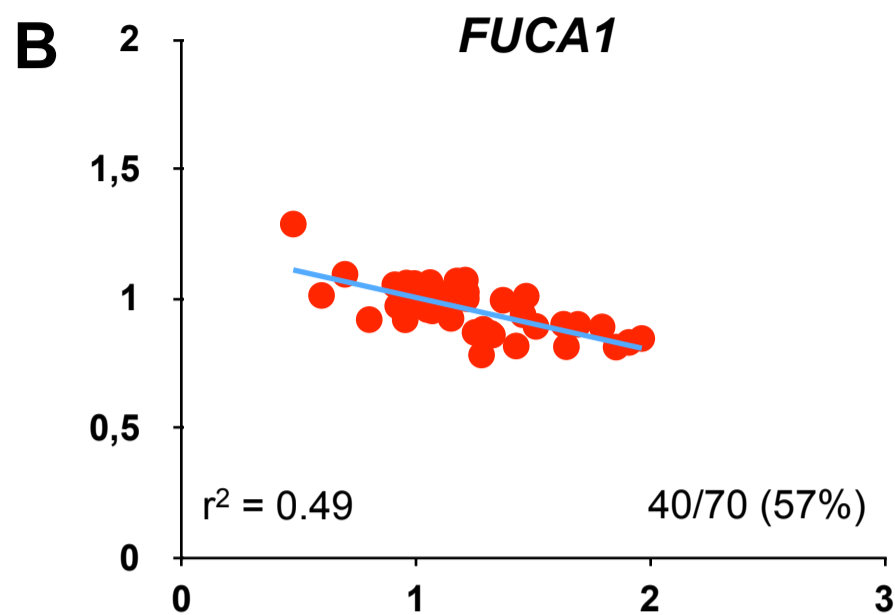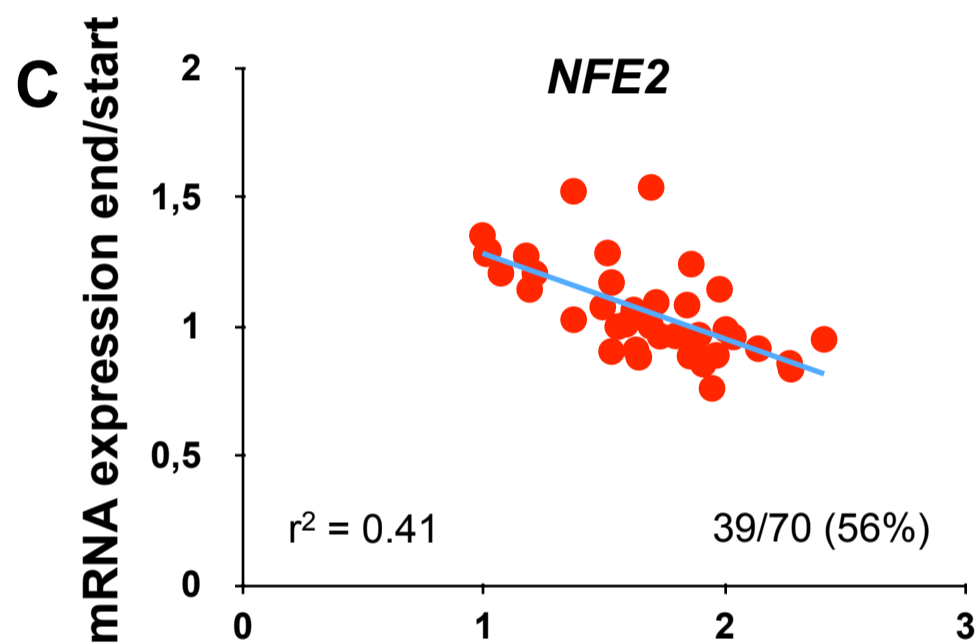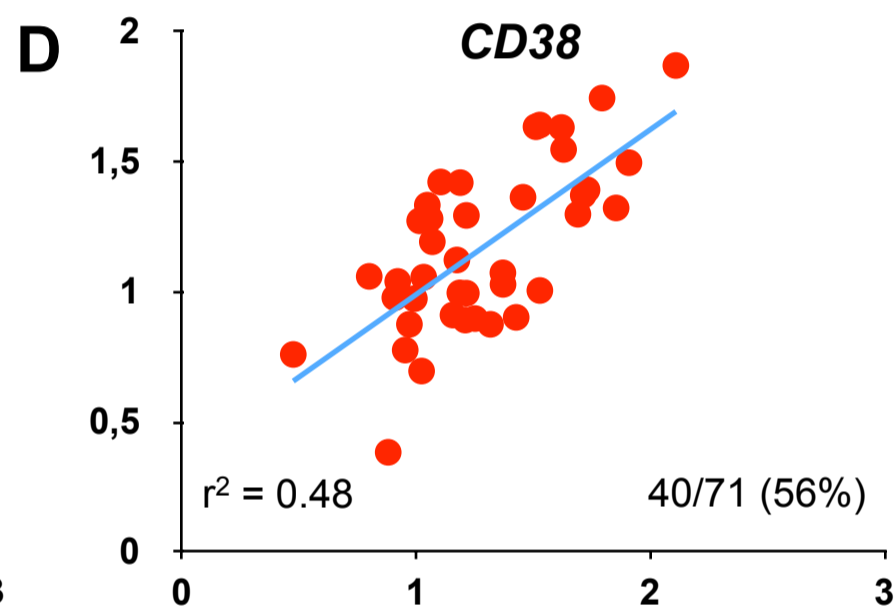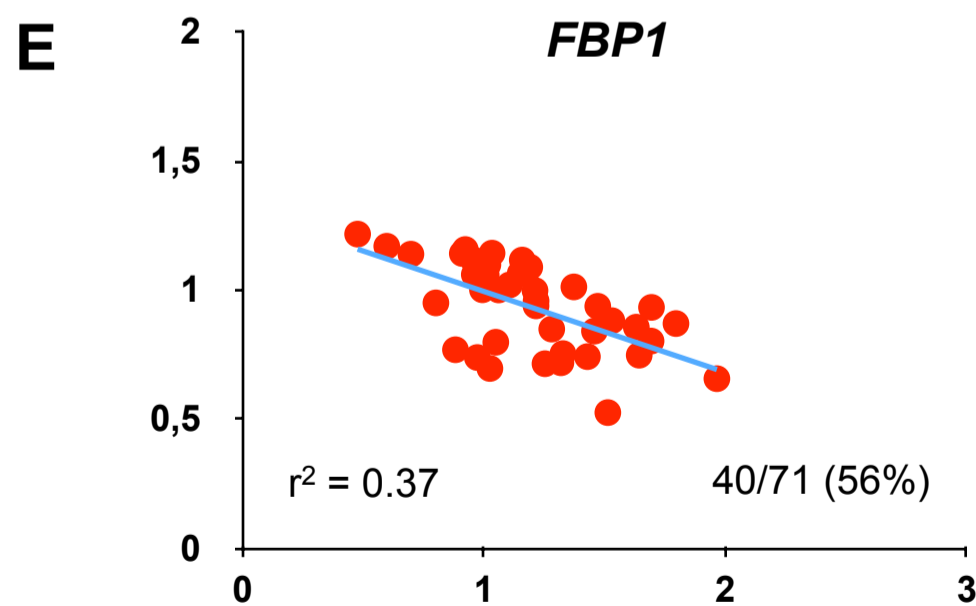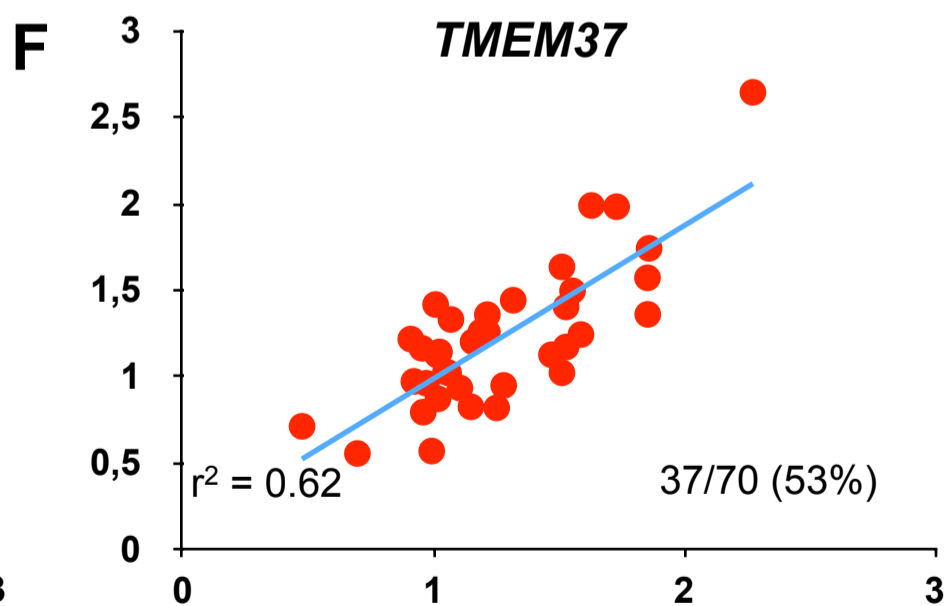

[25(OH)D3]end/start

Supplement: S2 Fig — RNA was isolated from PBMCs obtained from 70 or 71 participants of the VitDmet study before and after the 5-month vitamin D3 intervention. qPCR was performed to determine the relative changes of the expression of the VDR target genes CD274 (A), FUCA1 (B), NFE2 (C), CD38 (D), FBP1 (E) and TMEM37 (F) normalized by the reference genes B2M, GAPDH, HPRT1 and RPLP0. Linear regression analysis demonstrated the correlation between mRNA expression changes and alterations in the serum 25(OH)D3 levels of the participants. The number of selected study participants and the r2 correlation value are indicated. (PDF) [file pone.0124339.s005.pdf]

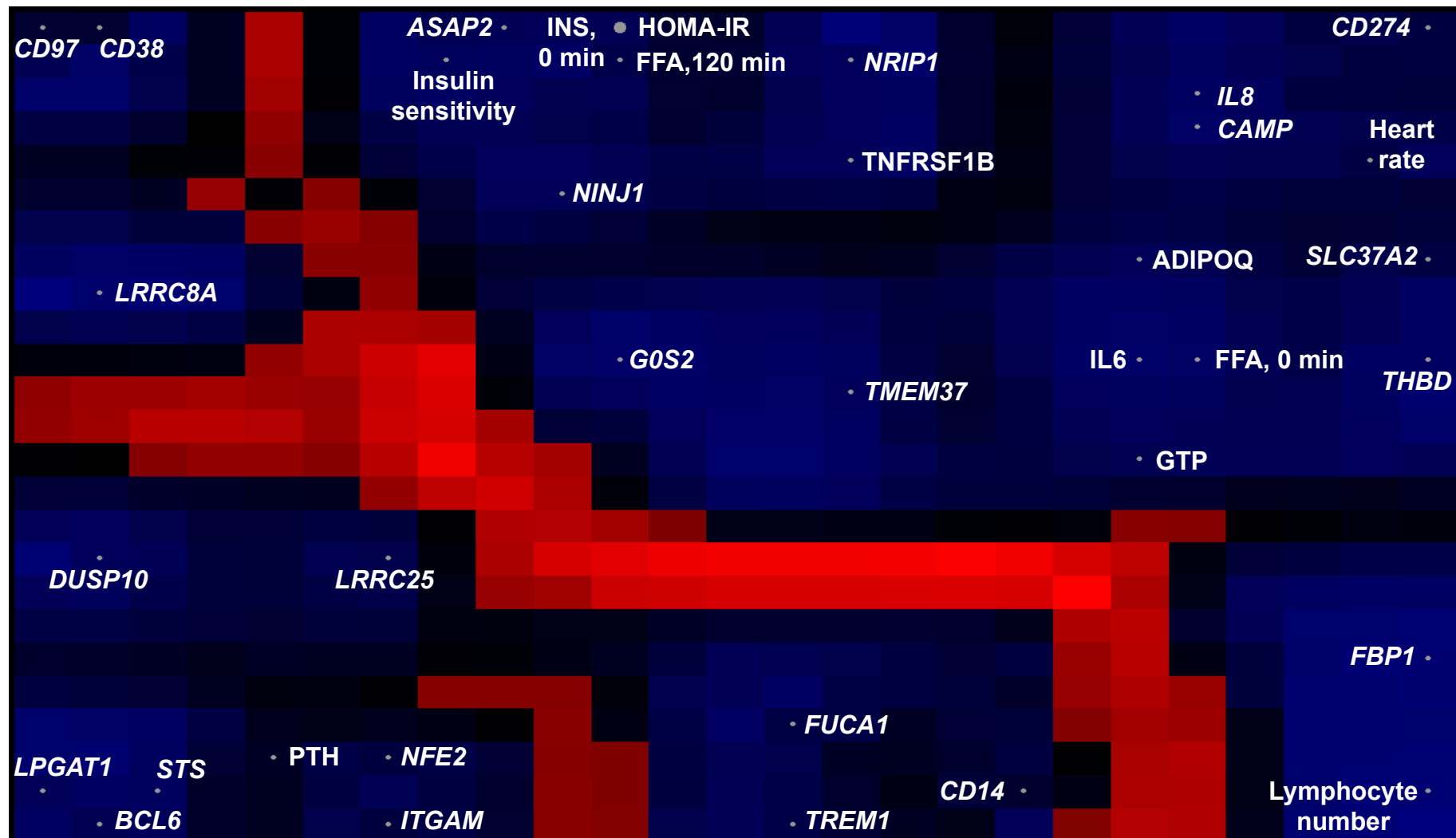

Supplement: S3 Fig — The correlation of the vitamin D3-dependent changes in the expression of 12 newly introduced VDR target genes, 12 previously analyzed [25] VDR target genes and physiological parameters is represented by a SOM of 25x25 units. The most relevant parameters cluster in the lower left corner of the map. The color of each unit is associated to the similitude of the surrounding units ranging from red (dissimilar) to blue (similar). (PDF) [file pone.0124339.s006.pdf]

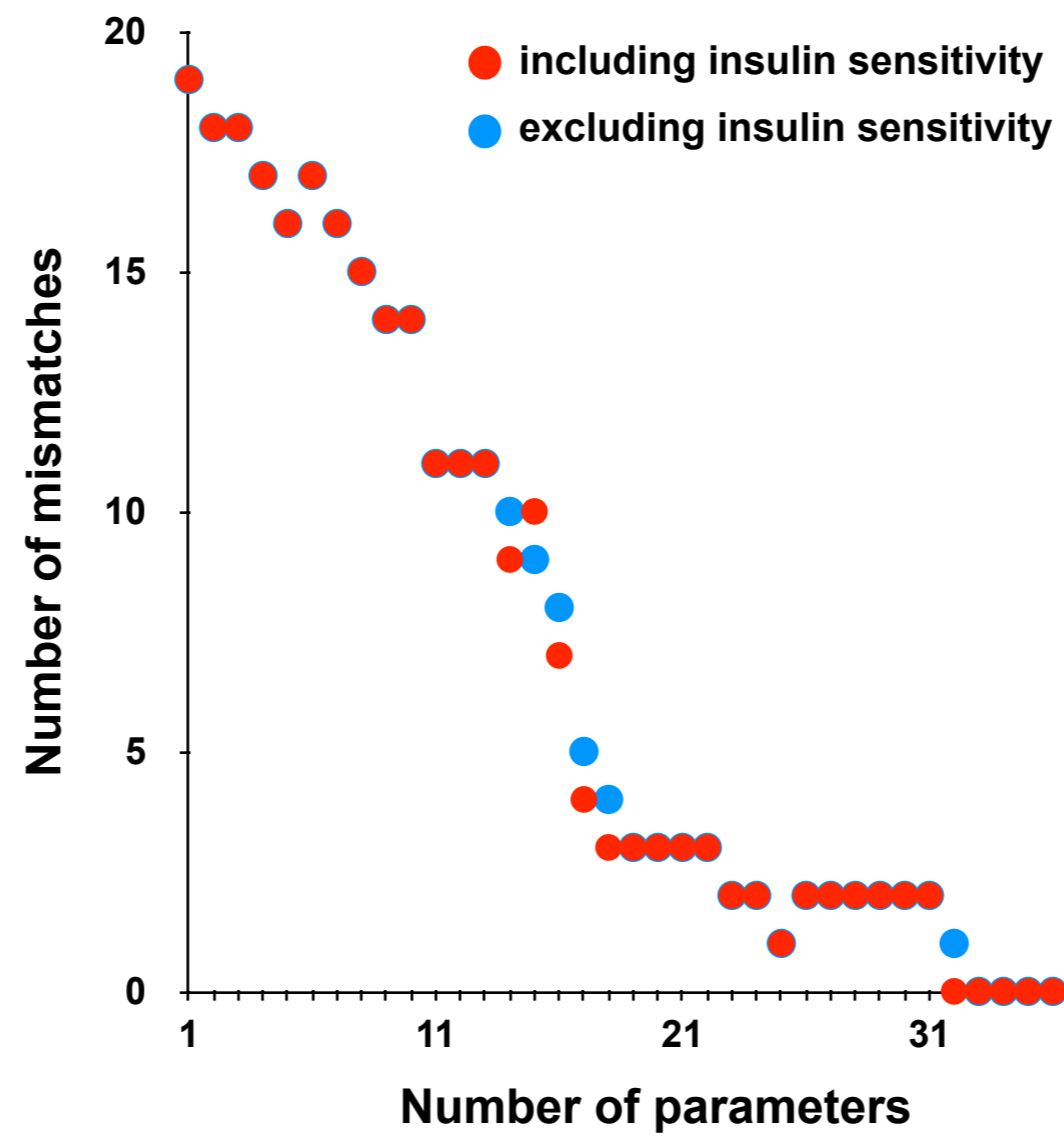

Supplement: S4 Fig — Starting in the order of the parameter relevance ranking (Fig 2B) the number of mismatches of segregating VitDmet participants into high and low responders was determined when increasing the number of used parameters (in reference to the classification shown in Fig 3). The first 17 parameters have to be included for a sufficiently accurate classification of the subjects. The analysis was done by including insulin sensitivity as 14th parameter (red) or by excluding it (blue). (PDF) [file pone.0124339.s007.pdf]

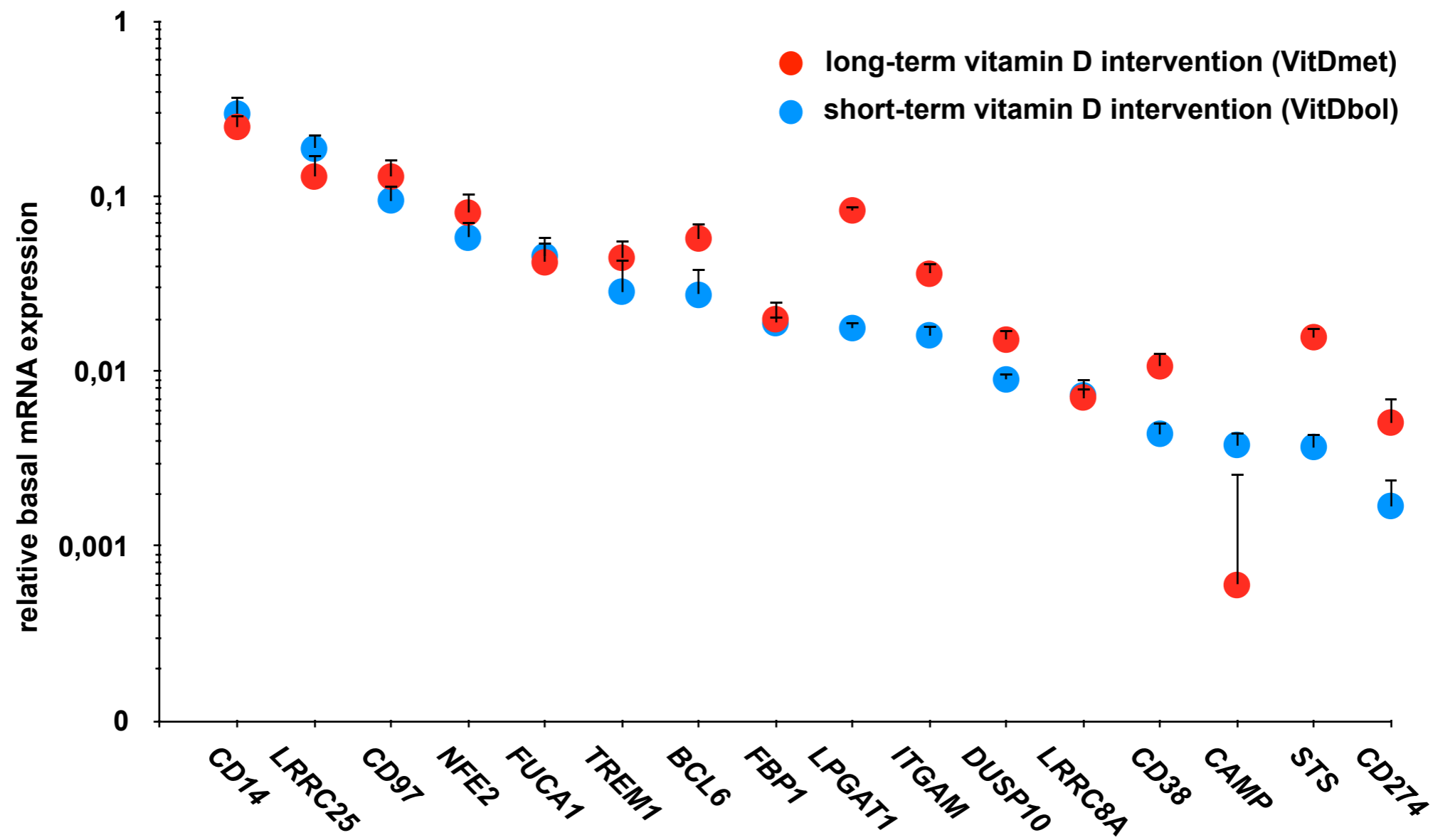

Supplement: S5 Fig — qPCR was used to determine relative mRNA expression of 16 most relevant VDR target genes in PBMCs isolated from 70 or 71 elderly pre-diabetic subjects at the begin of VitDmet study (red) or from 10 healthy adult subjects at the start of the of the VitDbol study (blue). Data points represent the means of gene expression and the bars indicate standard deviations. The genes were sorted by decreasing basal mRNA expression in VitDbol participants. (PDF) [file pone.0124339.s008.pdf]

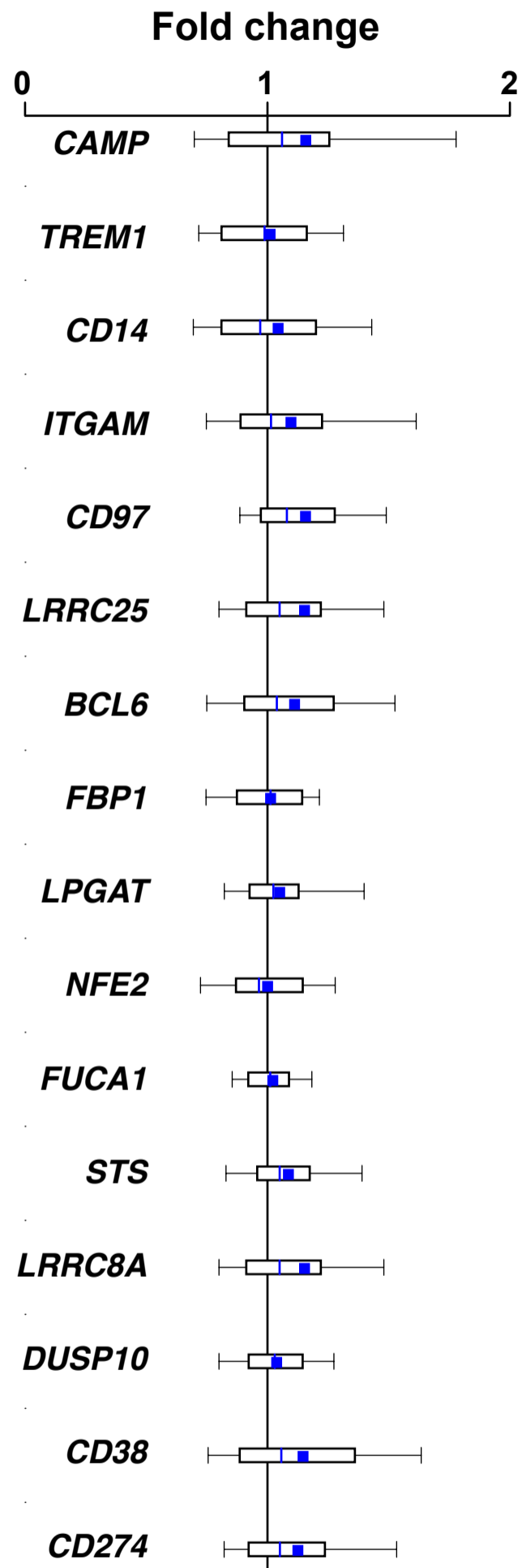

Supplement: S6 Fig — qPCR was used to determine the change in mRNA expression of 16 most relevant VDR target genes in PBMCs isolated from 70 or 71 elderly pre-diabetic subjects at the begin and the end of the 5-month vitamin D3 intervention trial (VitDmet). The top, fine central and bottom lines of the box plots represent the 75th, 50th and 25th percentiles, respectively, of the expression changes and the bars indicate standard deviations. The prominent blue central line within the box plots marks the means. The genes were presented in the same order as in Fig 4. (PDF) [file pone.0124339.s009.pdf]
